# Deciphering the Role of Selenoprotein M

**DOI:** 10.3390/antiox12111906

**Published:** 2023-10-25

**Authors:** Lance G. A. Nunes, Antavius Cain, Cody Comyns, Peter R. Hoffmann, Natalie Krahn

**Affiliations:** 1 Department of Cell and Molecular Biology, John A. Burns School of Medicine, University of Hawaii, Honolulu, HI 96813-5525, USA; 2 Department of Biochemistry and Molecular Biology, University of Georgia, Athens, GA 30602, USA; ac14754@uga.edu; 3 Department of Molecular Biophysics and Biochemistry, Yale University, New Haven, CT 06511-4902, USA

**Keywords:** selenocysteine, SELENOM, redox protein, selenoprotein, oxidoreductase

## Abstract

Selenocysteine (Sec), the 21st amino acid, is structurally similar to cysteine but with a sulfur to selenium replacement. This single change retains many of the chemical properties of cysteine but often with enhanced catalytic and redox activity. Incorporation of Sec into proteins is unique, requiring additional translation factors and multiple steps to insert Sec at stop (UGA) codons. These Sec-containing proteins (selenoproteins) are found in all three domains of life where they often are involved in cellular homeostasis (e.g., reducing reactive oxygen species). The essential role of selenoproteins in humans requires us to maintain appropriate levels of selenium, the precursor for Sec, in our diet. Too much selenium is also problematic due to its toxic effects. Deciphering the role of Sec in selenoproteins is challenging for many reasons, one of which is due to their complicated biosynthesis pathway. However, clever strategies are surfacing to overcome this and facilitate production of selenoproteins. Here, we focus on one of the 25 human selenoproteins, selenoprotein M (SELENOM), which has wide-spread expression throughout our tissues. Its thioredoxin motif suggests oxidoreductase function; however, its mechanism and functional role(s) are still being uncovered. Furthermore, the connection of both high and low expression levels of SELENOM to separate diseases emphasizes the medical application for studying the role of Sec in this protein. In this review, we aim to decipher the role of SELENOM through detailing and connecting current evidence. With multiple proposed functions in diverse tissues, continued research is still necessary to fully unveil the role of SELENOM.

## 1. Introduction

Selenium acts as a double-edged sword: an essential micronutrient for humans that becomes toxic in excess [1]. Selenium is found in both organic (selenomethionine, selenocysteine (Sec)) and inorganic (selenite, selenate) forms, of which the latter is found to be more toxic to humans [2,3]. Sec, the 21st natural amino acid, is biosynthesized on its tRNA to convert inorganic selenium to an organic form, readily used by cells for protein translation [3]. Humans have 25 selenoproteins (proteins containing Sec) that are responsible for cellular function (e.g., redox reactions, immune response, thyroid hormone metabolism). The inability to express these selenoproteins (due to selenium deficiency) have been associated with diseases including cancer, neurodegenerative diseases, Keshan disease, inflammatory bowel diseases, and diabetes [1,4,5]. Selenium supplementation has been proposed to prevent and treat some of these diseases; however, there are also data that show that excess selenium or overexpression of selenoproteins is connected to disease (e.g., diabetes, neurodegenerative diseases) [6,7,8,9,10]. These conflicting results suggest that maximizing selenoprotein production through selenium supplementation is not always a solution and we are missing key information to properly prescribe selenium [11]. Part of this gap is due to lack of understanding on the role that Sec has in the cellular function of selenoproteins [12].

The enhanced chemical reactivity of Sec has been an advantage when studying selenoproteins in vivo. Probes have been designed to specifically recognize Sec over cysteine, and radioactive selenium can be fed to cultures to visualize incorporation [13]. Studying selenoproteins in vitro, however, is more challenging. This is because they follow a translation path that is unique to all other proteins. Sec is encoded by a nonsense codon (UGA), but only in the presence of specific regulatory elements to designate Sec insertion instead of termination. Downstream of the specific UGA codon, in the 3′-untranslated region (UTR) of eukaryotes, is an mRNA hairpin known as the Sec insertion sequence (SECIS) element. This hairpin structure is highly conserved [14] for interaction with SECIS-binding protein 2 (SBP2) and a specialized elongation factor (eEFSec). eEFSec can discriminate the unique structure of tRNA^Sec^ from tRNAs for the other 20 amino acids, promoting elongation instead of termination [15,16]. Details of the translation mechanism involving these additional factors: SECIS element, SBP2, and eEFSec, are still not fully understood [17]. Moreover, the mechanism differs in each domain of life, adding to the complexity of feasibly overexpressing selenoproteins for detailed analysis of their cellular function [18].

Nevertheless, researchers have used clever strategies to unveil this looming question about selenoprotein function [13]. In this review, we have combined the studies on selenoprotein M (SELENOM) in an effort to connect what has been determined thus far and propose new directions that should be investigated. The extensive expression of SELENOM in the brain directed focus to this region initially; however, it is also found in other tissues. Therefore, researchers have started questioning the role of SELENOM throughout the body. The evidence presented suggests that SELENOM supports cellular growth and, specifically in the brain, has a neuroprotective role. Advancement in the technology to produce selenoproteins [19,20,21] opens the ability to further characterize SELENOM function.

## 2. Expression of SELENOM Is Widespread

**Figure 1:**
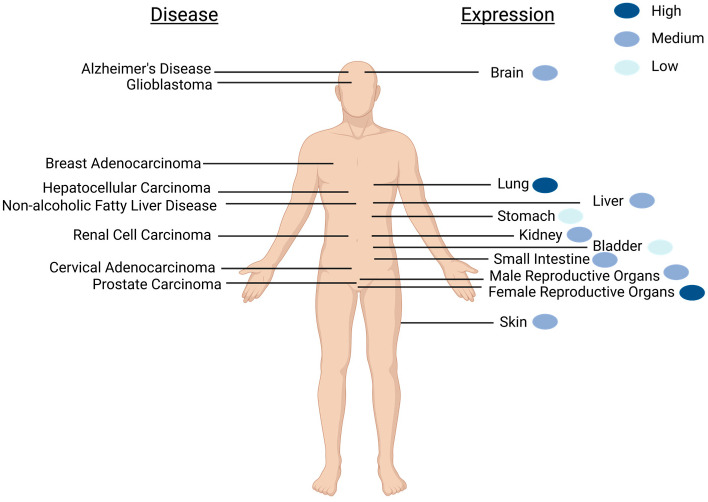
Schematic of SELENOM expression and SELENOM-related diseases as found in humans. Diseases that correlate with aberrant SELENOM expression are shown on the left, while the location of major organs with detectable SELENOM expression is on the right. Relative expression levels of healthy individuals are portrayed with blue-colored dots. The widespread expression of SELENOM is not limited to what is depicted. Created with BioRender.com.

SELENOM is widely expressed throughout the body (e.g., heart, lung, kidney, stomach, small intestine, skin, testis, uterus, ovary, and brain), but not expressed in all tissue types (e.g., muscle and thymus) [22,23] (Figure 1). In the brain, SELENOM expression is extensive, detectable in multiple brain regions including the olfactory bulb, cortex, hippocampus, hypothalamus, brain stem, cerebellum, and cerebellar cortex lysates [23,24]. Immunohistochemistry staining for SELENOM distribution in mice coronal brain sections revealed SELENOM localization in multiple brain regions including the periventricular and arcuate nuclei of the hypothalamus; the ventral tegmental area; red nucleus; the CA1, CA2, and CA3 regions of the hippocampus; the medial septum; and the granular, Purkinje, and molecular layers of the cerebellum [24]. This high expression level of SELENOM in multiple brain regions suggests an important role in brain function.

A global knockout of SELENOM in mice did not result in adverse cognitive or motor defects as one may have expected. Instead, metabolic dysregulation caused by diminished hypothalamic leptin signaling was observed [24,25]. Given that most of SELENOM is found in GABAergic cells, this observation made sense. However, one thing to consider when studying SELENOM in mice is that human expression levels are much lower and the distribution of selenoproteins differ between the organisms [26]. For example, mice have high expression levels of GPx1, GPx4, SELENOF, SELENOK, SELENOM, SELENOS, and SELENOW, while SELENOW and SELENOF are the highest expressed in humans [26]. Since the mechanisms by which many selenoproteins function are still not clear, we cannot rule out whether another selenoprotein in the brain compensates for cognitive and motor function in the absence of SELENOM.

Beyond the brain and examining the entirety of the human body, the Human Protein Atlas (https://www.proteinatlas.org/ENSG00000198832-SELENOM, accessed 14 March 2023) shows that generally SELENOM is expressed in the cytoplasm but localizes to the perinuclear region and nucleoplasm. SELENOM is also highly expressed in the thyroid gland, lungs, and female reproductive organs. The glandular system is most prominent for mRNA expression, with exocrine glandular cells expressing the highest levels. There is low cancer specificity for SELENOM; however, its expression in renal cell carcinoma (RCC) is unfavorable and used as a prognostic marker, discussed in a later section.

## 3. Elucidating SELENOM Function from Structure

### 3.1. SELENOM Is Structurally Close to Thioredoxin

**Figure 2:**
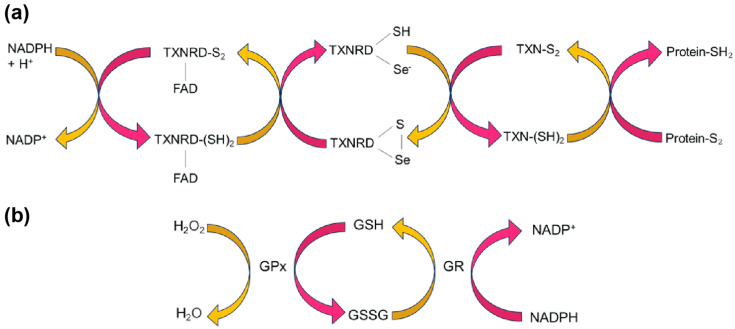
Oxidoreductase pathways. (**a**) The thioredoxin (TXN) cycle illustrates how TXNRD, an essential selenoprotein, catalyzes the reduction of oxidized thioredoxin (TXN-S_2_) using NADPH as an electron donor. Reduced thioredoxin (TXN-(SH)_2_) plays a critical role in oxidizing proteins involved in cellular redox homeostasis, influencing processes like DNA synthesis, antioxidant defense, and apoptosis. (**b**) In the GPx pathway, GPx catalyzes the reduction of peroxides, including hydrogen peroxide (H_2_O_2_), using reduced glutathione (GSH) as an electron donor. The resulting oxidized glutathione (GSSG) can be converted back to GSH through the action of glutathione reductase (GR), ensuring a continuous supply for cellular antioxidant defense. From activity assays, SELENOM could play a role in reducing peroxides like GPx.

As with elucidating the function of a newly discovered protein, when studying a new selenoprotein, an initial step is to scan the structure space for similar proteins that have been previously characterized. Structurally, the closest relative to SELENOM is thioredoxin [27]. Thioredoxins are oxidoreductases with a defined thioredoxin (TXN)-fold identified by a two-layer α/β/α sandwich with a βαβββα secondary structure. Moreover, they harbor a CXXC active-site motif, where X can be any amino acid [28]. Through this CXXC motif, TXNs catalyze the reduction of disulfide bonds as part of a catalytic cycle involving thioredoxin reductase (TXNRD) or through activation by reaction oxygen species (ROS) [29,30] (Figure 2a). In SELENOM, the CXXC motif is found as CXXU, where U refers to Sec [22]. The similar chemistry between C and U suggests that this motif also serves as a redox center and SELENOM participates as an oxidoreductase.

The redox active amino acids (C or U) in the CXXC/U motif are surface-accessible in SELENOM. This differs from TXN, but is seen in protein disulfide isomerases (PDIs). PDIs are also oxidoreductases that typically consist of two catalytic TXN-like domains (containing the CXXC motif), separated by two non-catalytic TXN-like domains. However, some PDIs only contain a single catalytic domain [31,32], which is analogous to what is observed in the structure of SELENOM. Furthermore, the position of the CXXU motif is located between the C-terminus of strand β1 and *N*-terminus of helix α1, which compares to what is found in both TXNs and PDIs [27].

### 3.2. SELENOM Defines the New Thioredoxin Family

**Figure 3:**
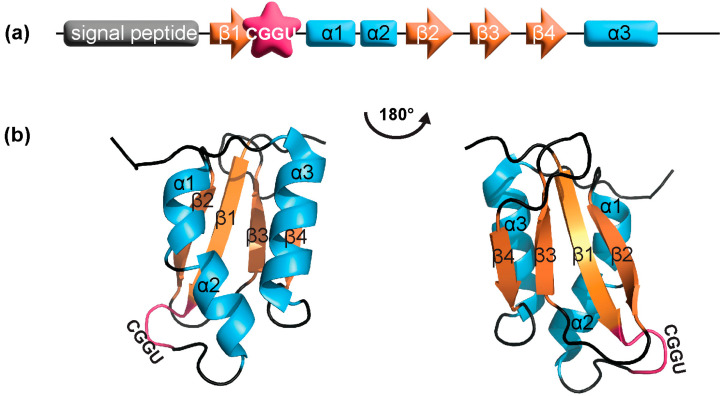
SELENOM (**a**) domain structure and (**b**) NMR structure (PDB: 2A2P). The first 25 residues are the endoplasmic reticulum signal peptide, which was removed for protein expression. Residues 25–34 and 121–145 are not shown in the structure due to high flexibility. The CXXU motif is shown in magenta, α-helices in cyan, and β-strands in orange.

Another structural homolog found for SELENOM is SELENOF (previously named Sep15) [22,33]. Although their sequence identity is only 31%, SELENOF shares multiple regions of significant sequence identity to SELENOM [34]. The major similarity that distinguishes SELENOM and SELENOF from other TXN families is its unique TXN-like fold. Its central α/β domain, composed of three α-helices (α1-α3) and a mixed parallel/anti-parallel four-stranded β-sheet (β1-β4), represents the most basic TXN-like fold [35] (Figure 3). The CXXU motif located within this unique TXN-like fold is also unlike other oxidoreductases. While X refers to any amino acid, only certain amino acids are typically found in nature. These include GP, GH, and PH, found in TXNs, PDIs, and disulfide oxidases, respectively [27]. SELENOM has the sequence motif CGGU, which has only been observed thus far in SELENOF as CGU [27].

In addition to a defining TXN-like fold, both SELENOM and SELENOF have other structural features that group them into a separate subfamily of TXNs. The conserved proline at the *N*-terminus of strand β3 is typically found in the cis-conformation [29], while in SELENOM and SELENOF, it is in the trans-conformation [28]. Furthermore, these proteins are missing a charge pair that in TXNs and PDIs are involved in proton transfer [30]. The functional importance of these structural differences remains unclear and is still under study.

### 3.3. SELENOM Does Not Bind UGGT

Among the many similarities between SELENOF and SELENOM, there are differences that potentially separate their functions. These differences lie at the termini. Both selenoproteins have an *N*-terminus that contains an ER-signaling sequence, which is subsequently cleaved during protein maturation. In SELENOF, an elongated cysteine-rich extension follows the signaling sequence prior to strand β1, while in SELENOM, this is a short extension. Furthermore, the C-terminus of SELENOM is a flexible extension, but it is short and unstructured in SELENOF [27]. The cysteine-rich extension at the *N*-terminus of SELENOF is known to mediate a high-affinity complex with the folding sensor of the calnexin cycle-UDP-glucose:glycoprotein glucosyltransferase (UGGT) [34,36]. The function of this binding interaction is not fully investigated, though it is suggested to be a PDI co-factor, assisting UGGT in assessing misfolded glycoproteins [27]. The absence of this cysteine-rich region in SELENOM confirmed that UGGT is not an interacting partner for SELENOM [34].

## 4. Elucidating SELENOM Function from Observed Activity

While structurally SELENOM resembles other oxidoreductases, some of the defining functional features from these oxidoreductases are not shared with SELENOM. Therefore, experimental studies have been employed to investigate this further. Similar to the structural comparisons above, functional comparisons with predefined oxidoreductase assays were investigated.

### 4.1. Glutathione Peroxidase Activity

Glutathione peroxidase (GPx), a member of the selenoprotein family, serves as a critical antioxidant enzyme, and its functionality is intricately linked to its molecular structure. The enzyme’s catalytic efficiency is largely attributed to a Sec residue at its active site, which enables GPx to rapidly reduce hydrogen peroxides and lipid peroxides to water and alcohols, respectively. Peroxides oxidize GPx, which reacts with reduced glutathione (GSH). This oxidizes glutathione (GSSG), converting GPx back to its active (reduced) state for reaction with another peroxide. Glutathione reductase subsequently regenerates GSH from GSSG using NADPH as the reducing agent to complete the cycle (Figure 2b). This integrated antioxidant system, facilitated by the high reactivity of selenium and efficient enzyme kinetics, allows for rapid detoxification and maintenance of cellular redox balance [37]. Furthermore, the enzyme’s multimeric architecture—existing as either a dimer or tetramer—enhances its kinetics and enables substrate-specific channels that guide peroxides toward the Sec active site for catalysis [38].

To test for GPx activity, a GPx assay is established, which monitors the conversion of reduced glutathione (GSH) to oxidized glutathione (GSSG) while reducing hydrogen peroxide (H_2_O_2_) or other peroxides to water or alcohol. This enzymatic reaction cycle involves GPx and glutathione reductase, which helps convert GSSG back to GSH, thereby maintaining a pool of available GSH for the GPx reaction. The assay is designed to monitor the rate of NADPH oxidation to NADP+ as a direct measure of GPx activity. SELENOM has been shown to have GPx activity in vitro [39,40] and also shown to reduce ROS in HEK293T cells. Replacing Sec in the CXXU motif with a Cys, significantly increased the ROS present, presumably decreasing the activity of the enzyme. This shows that efficient reduction of ROS relies on the presence of Sec, due to the difference in chemistry between the two amino acids [37].

### 4.2. Thioredoxin Activity

TXN is a small redox-active protein known for its pivotal role in cellular redox homeostasis and other cellular processes, such as DNA synthesis and signal transduction [41]. It acts through the reduction of disulfide bonds in target proteins, often initiated by reduction of TXN by other proteins like TXNRD [42]. This catalytic cycle of reducing disulfide bonds with TXN, TXNRD, and NADPH is known as the TXN system (Figure 2b). Moreover, in the presence of NADPH and TXNRD, TXN activity can be measured on a target protein such as insulin. Based on the proposed similarity in disulfide bond formation function of TXN and SELENOM, this assay was performed on hypothalamic tissues and cells that are SELENOM deficient. Through this, a decrease in the TXN activity was observed, although levels of TXN and TXNRD were not affected. Further evidence showed TXN activity in SELENOM-immunoprecipitated samples suggesting that indeed SELENOM has intrinsic TXN activity and plays a crucial role in enhancing the TXN system’s antioxidant activity. This highlights SELENOM’s potential influence on regulating energy balance in hypothalamic tissues and defending cells from oxidative stress [25].

### 4.3. Absence of Thioredoxin Reductase Activity

Another oxidoreductase assay that has been established is the TXNRD assay. TXNRD is a selenoprotein involved in reducing the oxidized form of TXN. This occurs through the Sec-containing active site located at the C-terminus of the enzyme. Sec is critical to the enzyme’s efficiency because of its higher nucleophilicity compared to a sulfur-containing Cys. Thus, the selenium atom in Sec is directly involved in the enzyme’s catalytic mechanism, contributing to rapid and efficient reduction reactions. In addition to the Sec requirement for TXNRD activity, the enzyme also relies on its FAD cofactor to reduce TXN. In TXNRD, FAD is tightly, yet non-covalently, anchored within a specific segment of the protein, commonly referred to as the FAD-binding domain [43]. This domain is pivotal for enabling redox reactions by initially accepting electrons from NADPH. FAD serves as a redox-active cofactor, facilitating the transfer of electrons, pivotal for TXNRD’s role in reducing oxidized TXN (Figure 2b). In assays that assess TXNRD activity, a common approach is to measure the increase in DTNB (5,5′-dithiobis-(2-nitrobenzoic acid) levels, indicative of the enzyme’s capability to reduce TXN, which acts as an intermediary substrate in this process [44].

TXNRD activity was also assessed for SELENOM in hypothalamic tissues and cells. Initial observations found a decrease in TXNRD activity in SELENOM-deficient cells but not tissues. However, upon further investigation with SELENOM-enriched samples, no increase in TXNRD activity was observed [25]. This result is not completely surprising given that structural similarities connect SELENOM to TXN rather than TXNRD. Therefore, as a parallel to the TXN system, SELENOM may also be part of a catalytic cycle for the reduction of disulfide bonds in proteins.

## 5. Elucidating SELENOM Function from Binding Partners

### 5.1. Thioredoxin Interacting Protein

Thioredoxin interacting protein (TXNIP), also known as thioredoxin-binding protein 2, is a major mediator in the TXN antioxidant system. TXNIP interacts with the CXXC motif of TXN, blocking its potential to scavenge ROS. This in turn increases the ROS levels in the cell and induces apoptosis [45]. TXNIP is also known to be a negative regulator of mTOR-dependent signaling [46], with implications that it modulates hypothalamic leptin signaling [47,48]. Micro-array analyses of SELENOM-deficient mHypoE-44 cells and hypothalamic tissue identified a downregulation of TXNIP but no change in expression levels for TXN and TXNRD [25]. This suggests that the reduction in TXNIP is a compensatory response adapting to the absence of SELENOM. Furthermore, an observed decrease in the TXN activity (but not the TXNRD activity) suggests that SELENOM contributes to the hypothalamic TXN system [25]. The CXXU motif in SELENOM and corresponding TXN activity implies an interaction with TXNIP similar to TXN. It follows that SELENOM and TXNIP work together to maintain cellular homeostasis.

**Figure 4:**
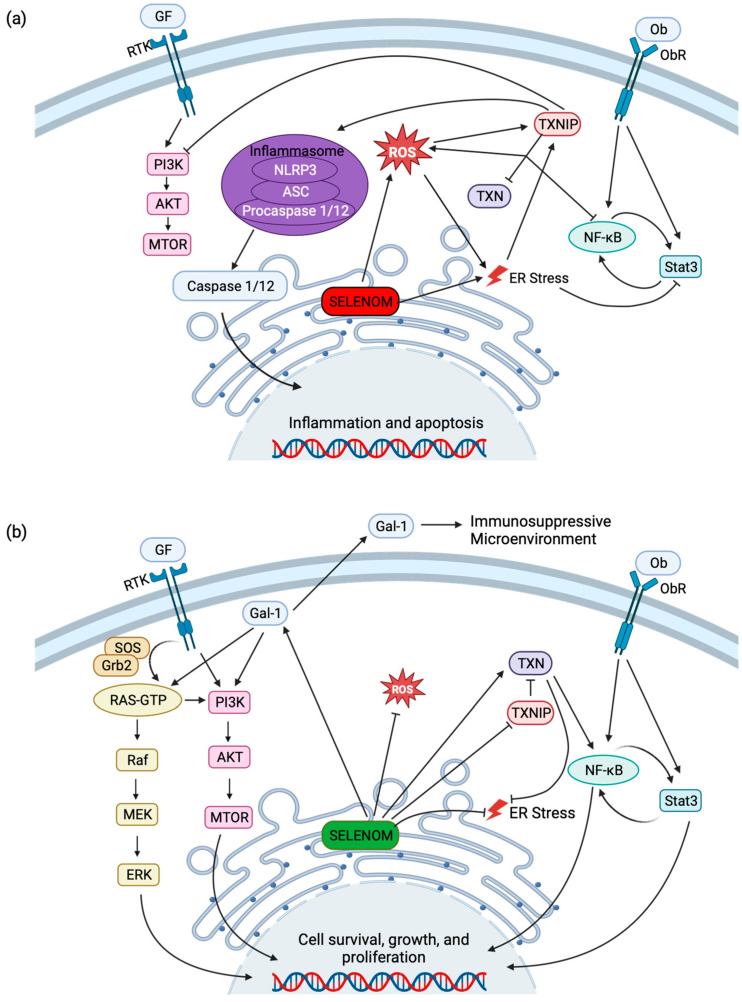
SELENOM and related signaling pathways. (**a**) Low levels of SELENOM (red) are correlated with an increase in ROS and may induce ER stress. Elevated ROS and ER stress increase TXNIP activity, downregulating TXN activity, thereby resulting in the suppression of NF-κB and increase in ROS. TXNIP suppresses cellular growth and survival by inhibition of PI3K/AKT/mTOR signaling and promotes the caspase-mediated induction of inflammatory and apoptotic genes by activating the NLRP3 inflammasome. (**b**) High levels (green) of SELENOM decrease ROS generation and promote TXN activity by the suppression of TXNIP. Promotion of transcription factors NF-κB and Stat3 by leptin signaling and TXN activity as well as SELENOM/Gal-1 activation of PI3K/AKT/mTOR and Ras/Raf/MEK/ERK signaling results in the transcription of pro-survival, growth, and proliferation genes. Gal-1 also is secreted in the extracellular space as an anti-inflammatory signaling molecule. Created with BioRender.com.

This has been observed in neurons where SELENOM reduced neuronal apoptosis in response to oxidative stress through regulating cytosolic Ca^2+^ release from the ER [40]. TXNIP, on the other hand, is induced in high-oxidative environments, activating cellular apoptosis [49]. Independent observations found increased levels of TXNIP in neurodegenerative disease [49] and low levels of SELENOM [50,51]. In the absence of SELENOM, ER stress increases, promoting the expression of TXNIP and neuronal apoptosis and inflammation (Figure 4a) [49,52,53]. In cancers, the opposite has been observed, wherein TXNIP levels are lowered and SELENOM levels are higher [49]. The high levels of SELENOM reduce the ROS species, lowering the expression of TXNIP and promoting cellular growth (Figure 4b).

### 5.2. Galectin-1

To identify other protein partners that could be involved in neuroprotection, a yeast two-hybrid system was used to screen a human fetal brain cDNA library. This resulted in the identification of galectin-1 (Gal-1) as an interactive binding partner of SELENOM [54]. Gal-1 is differentially expressed across many tissues and is suggested to have a wide range of biological activity both intra- and extracellularly. With respect to the brain, Gal-1 is found across the central and peripheral nervous systems, involved in nerve development and axon regeneration. Specifically, oxidized Gal-1 has been shown to promote neurite outgrowth and enhance axonal regeneration in nerves [55]. The detailed interaction of SELENOM and Gal-1 is not yet described; however, we know that Sec is not required for binding since the pull-down assay was performed with a Sec-to-Cys mutation in SELENOM. It is possible, however, that if SELENOM is involved in the oxidation of Gal-1, the presence of a Sec would be more efficient in the oxidoreductase activity than with Cys.

### 5.3. Cytoplasmic Actins

The presence of the CXXU motif in SELENOM, as mentioned above, suggests oxidoreductase activity like TXN. TXN activity functions through the formation of disulfide bonds using its CXXC motif. To search for proteins that interact with the proposed catalytic site of SELENOM, purified protein was incubated with cancer cell lysates (HT-1080 and MCF-7), followed by reduction to release proteins interacting through a disulfide bond. The multi-step translation path for Sec insertion challenges recombinant selenoprotein production [13]. Therefore, the search for interacting proteins was performed with a Sec-to-Cys mutation in SELENOM. A similar strategy has been used previously to screen for protein partners with other selenoproteins (SELENOV and SELENOW) [56,57]. This experiment led to the detection of cytoplasmic actin 1 and 2 (β- and γ-actin), key proteins in adhesion, migration, polarization, and mitosis of cells [58]. Interaction via a disulfide suggests that SELENOM is involved in reducing oxidized actin, playing a role in regulating the actin cytoskeleton. These cellular processes are involved in the metastasis of tumors and further enhance the explanation for the role of increased SELENOM that is observed in some cancers [12].

### 5.4. Osteoblast Colocalization

SELENOM has been shown to have a role in bone development as well as in cartilage formation. In situ hybridization studies on chickens detected SELENOM mRNA expression in the condensed mesenchyme, a cluster of cells that later differentiate into bone [59]. Furthermore, in situ hybridization on embryonic day 16.5 mice revealed SELENOM expression in the vertebrae, maxilla, mandibula, trabecular and periosteum long bones, and the olfactory epithelium. SELENOM expression was also observed in the teeth and bones of postnatal day 4 mice [60]. To further the involvement of SELENOM in bone formation, SELENOM expression was found to colocalize with markers for osteoblasts, such as the early osteoblast marker *Bglap* [59,60]. Deficiency in selenium leads to a reduction in SELENOM expression by almost 70% in chicken cartilage tissues [60]. While the evidence suggests that SELENOM functions in bone development, the understanding of mechanisms and key players involved in this process is still a mystery. Knowledge of this can bring new insights into healing fractured or broken bones and to combat developmental or adult bone disorders.

### 5.5. Hypothalamic Leptin Signalling

SELENOM is found to be highly expressed in several regions of the brain. In efforts to identify its role, a global knockout of the SELENOM gene (SelenoM^−/−^) in mice was generated. As these mice developed, they were found to have increased body weight, elevated white adipose tissue levels, and reduced leptin response [24]. Leptin is a hormone that acts within the hypothalamus to regulate energy balance via signaling pathways to suppress appetite or increase energy expenditure. When the hypothalamus does not respond normally to these leptin signals (i.e., leptin resistance), this results in obesity. One established cause of leptin resistance is continual stress in the ER, which can be caused by either accumulation of misfolded proteins, Ca^2+^ depletion, or a combination of the two [61,62]. The proposed oxidoreductase activity of SELENOM from its CXXU motif suggests its function in the maturation of proteins through disulfide formation. Therefore, without SELENOM present in the mouse, misfolded proteins could accumulate, inducing ER stress. Moreover, in vitro studies found SELENOM to be involved in Ca^2+^ homeostasis. Overexpression of SELENOM in HT22 and C8-D1A cell lines reduced changes in cytosolic Ca^2+^ levels that would otherwise increase during oxidative stress. The increased basal levels observed in the knock-down suggest that Ca^2+^ regulation is impaired preventing any response to increased Ca^2+^ levels [40]. This was further corroborated with SELENOM shRNA-expressing hypothalamic cells (silencing expression of the protein), where Ca^2+^ levels remained unchanged under leptin treatment [25]. These data propose that SELENOM facilitates communication between the leptin receptor and ER proteins involved in Ca^2+^ signaling. A hypothesis based off the mechanism of other selenoproteins [63,64], is that this is accomplished by indirectly reducing the thiol groups on ER Ca^2+^ channels or pumps.

## 6. SELENOM Implications in Disease

To further interrogate the localization and function of SELENOM, we investigated the diseases associated with aberrations in normal SELENOM expression. In this section, we summarize key findings connecting SELENOM to diseases such as Alzheimer’s dementia (AD), non-alcoholic fatty liver disease (NAFLD), and cancers. These are the most studied and well-known diseases that SELENOM has been associated with.

### 6.1. Alzheimer’s Disease

Alzheimer’s dementia (AD) is a neurodegenerative disease that results in progressive loss of cognition and ultimately dementia. There are three known genes wherein mutations cause the development of AD: amyloid precursor protein (APP), presenilin-1 (PSEN-1), and presenilin-2 (PSEN-2) [65]. PSEN-1 and -2 are components of γ-secretase, which is responsible for the cleavage of APP to produce amyloid-β (Aβ). Mutations in human PSEN genes alter the cleavage of APP by γ-secretase, resulting in a higher ratio of Aβ isoform Aβ_42_ and the development of early onset familial AD (eFAD) [66,67]. eFAD makes up a small percentage (less than 5%) of AD in humans caused by a single genetic mutation [68]. Although single gene mutation mouse models of AD typically present with just one hallmark of AD and lack other pathologies routinely observed in patients with AD, they still provide a useful tool to study such a complex, typically multigenetic and multifactorial disorder whose etiology remains largely unclear.

The connection of selenoproteins to AD was found through a specific knockout of *Trsp* (the gene for tRNA^Sec^) in mouse neurons. This disrupts the ability to express selenoproteins (including SELENOM) and induces a neurodevelopmental and neurodegenerative phenotype affecting the cerebral cortex and hippocampus [50]. The similar yet milder phenotype found in *Gpx4*-deficient mice suggests that GPx4 could be one of the major selenoproteins involved [69]. However, there must be at least one additional selenoprotein participating in neuroprotection and neural development to reach the extent of the phenotype observed through *Trsp* knockout. In a mouse model of AD that expresses human mutant PSEN-2 (N141I), SELENOM expression was found to be downregulated [51]. These data propose that SELENOM may be one of the missing selenoproteins involved in neurodegeneration. The pathogenicity of AD has been correlated to the secretase-mediated generation of Aβ_42_ peptides deposited at neuritic plaques [70]. Selenium has been found to decrease the γ-secretase activity in mice through activation of the extracellular-signaling-regulated kinase (ERK) pathway [71]. Overexpression of human SELENOM in addition to selenium supplementation further decreased the γ-secretase activity, while a decrease in α-secretase activity and an increase in β-secretase activity was also observed. The combination of these changes contributed to a decrease in Aβ_42_ production, lowering the progression of AD [72]. Being that SELENOM is a selenoprotein, the natural question to ask is whether the Sec residue is involved in protein function. In a study using HEK293 cells, cotransfection of Aβ_42_ with either SELENOM or SELENOM’ (a SELENOM variant in which the Sec is replaced with Cys) resulted in a significant reduction of Aβ_42_ aggregates in vitro [73]. This suggests that SELENOM prevention of Aβ_42_ aggregates is not influenced by the presence of Sec. Additionally, cotransfection of HEK293 cells with Aβ_42_ and an empty vector produces abnormal mitochondrial localization near Aβ plaques and swollen morphology. Addition of either SELENOM or SELENOM’ restores mitochondrial localization and morphology by preventing an increase in intracellular ROS [73]. Therefore, SELENOM may serve a neuroprotective role through exerting antioxidant activity in the brain.

Another hallmark of AD is the development of neurofibril tangles (NFT) due to the aggregation of hyperphosphorylated tau. NFTs purified from AD brains are enriched with hyperphosphorylated tau [74]. It has been shown that this hyperphosphorylation reduces tau’s ability to bind to microtubules, promoting self-aggregation and formation of NFTs in the cytoplasm of neurodegenerative disease cells [75,76,77]. Thus, uncovering mechanisms that reduce tau phosphorylation is attractive for the purposes of preventing NFT formation. ERK pathway activation via SELENOM overexpression and selenium supplementation led to decreased tau phosphorylation at three sites, Ser404, Ser202, and Thr231 [72]. Taken together, the data in this section indicate a connection of SELENOM expression with neuronal processes.

### 6.2. Cancer

SELENOM has also been studied in the realm of cancer biology with altered SELENOM expression observed in human tumor tissues. In some tumor types such as lymphoma, breast, fallopian tube, and ovarian cancers, SELENOM expression is decreased, while in others such as in parotid and uterine tumors, it is increased [1]. Human renal cell carcinoma (RCC) is a tumor type that has increased levels of SELENOM expression compared to normal kidney tissue. SELENOM can be used for prognosis with higher levels correlated to shorter overall patient survival [78]. This is suggested to be due to the role that SELENOM plays in cell survival; in vitro SELENOM silencing resulted in lower cell viability, and SELENOM overexpression produced increased cell viability. Moreover, SELENOM knockdown reduced the migratory capacity of two cell lines (CAKI-1 and 786O) derived from RCC [78]. For tumor cells to incur metastatic ability, they must achieve epithelial–mesenchymal transition (EMT). In the absence of SELENOM in CAKI-1 and 786O cells, key EMT-related genes were significantly downregulated, namely, *N*-cadherin, vimentin, β-catenin, and matrix metalloproteinase (MMP)-2 and MMP-9. Furthermore, the pathways involved in tumor growth and progression (PI3K/AKT/mTOR signaling) were also downregulated in the absence of SELENOM [78]. This demonstrates that SELENOM is positively correlated with the metastatic ability of RCC cells. RCC is one type of cancer that has been specifically investigated as a prognostic marker; however, other cell carcinomas require further studies [79].

The mitogen-activated protein kinase (MAPK) pathway is activated by selenium supplementation and SELENOM overexpression [72]. Specifically, the Raf/MEK/ERK pathway has been shown to be correlated with multiple cancer types such as in RCC, hepatocellular carcinoma (HCC), non-small cell lung cancer (NSCLC), melanoma, and papillary thyroid carcinoma [80]. ERK has been shown to promote cell migration, proliferation, and viability and has been associated with HCC progression. P38 and Jun *N*-terminal kinases, members of the two other MAPK pathways, have been associated with HCC development [81]. Moreover, higher SELENOM expression was discovered in hepatoma cell lines when compared to normal hepatocytes [82], and its overexpression in HCC liver tissues warranted its proposal as a putative marker for HCC [83]. Together, these reports elucidate another correlation between SELENOM and cancer progression.

A study in 2016 investigated the expression of six selenoproteins (SELENOH, SELENOK, SELENOM, SELENOS, SELENOV, and GPx6) in a variety of tumor cell lines: HT 1080 (fibrosarcoma), HepG2 (hepatocellular carcinoma), MCF7 (breast adenocarcinoma), A-172 (glioblastoma), HeLa (cervical adenocarcinoma), and DU-145 (prostate carcinoma). All six of the tumor cell lines investigated were found to express SELENOM mRNA at a relatively consistent level, while SELENOH and SELENOK levels varied more. In some cell lines they were expressed significantly more than SELENOM (HT-1080, HepG2, and HeLa), while in others, their expression levels were similar (MCF7, A-172, and DU-145). SELENOS, SELENOV, and GPx6 on the other hand were not detected in any of these six tumor cell lines [84]. Given the pro-growth attributes of SELENOM, its dysregulation may promote tumor growth and metastasis in multiple tissue types and forms of cancer.

### 6.3. Non-Alcoholic Fatty Liver Disease

The high prevalence of obesity worldwide poses a major risk for the development of NAFLD, the most common chronic liver disease type [85]. In NAFLD, patient health is complicated by abnormal lipid metabolism, mitochondrial dysfunction, oxidative stress, and lipid peroxidation [86]. NAFLD can develop from a high-fat diet (HFD) and is typically accompanied by an accumulation of lipid in the liver followed by lipotoxicity-induced hepatocyte inflammation and apoptosis [87]. SELENOM was recently connected to NAFLD utilizing an in vivo mouse model of HFD-induced NAFLD and an in vitro model of hepatocyte palmitic acid (PA)-mediated lipotoxicity [88]. In the in vivo model, a reduction in SELENOM mRNA and protein levels were accompanied by significantly higher body weight and increased levels of hepatic metabolism markers, liver weight, hepatic vacuolization, steatosis, nuclear atrophy, and overall liver degeneration including an increase in liver-fibrosis-related genes. Liver-specific knockout of SELENOM (Liv-SELENOM^−/−^) exacerbated these in vivo results. The in vitro model showed similar data, with reduced SELENOM mRNA and protein levels compared to non-PA-treated hepatocytes. Upon SELENOM overexpression, liver-fibrosis-related genes were restored [88]. These results demonstrate that HFD- and PA-mediated lipotoxicity downregulate SELENOM expression, and deletion of SELENOM worsens the development of HFD-induced NAFLD hepatic injury. Further laboratory and human studies have yet to be performed to confirm a connection between SELENOM and its potential to protect against hepatic injury.

Another observation in the NAFLD models was the higher levels of inflammatory markers such as TNF, interleukin (IL) 6, and IL1b inflammatory cytokines [88]. Chronic inflammation is a hallmark of NAFLD, which leads to cirrhosis, fibrosis, liver failure, and potentially hepatocellular carcinoma [89,90]. The connection to SELENOM was inferred when overexpression in the in vitro model restored expression levels of inflammatory markers and genes involved in oxidative stress and fatty acid oxidation (FAO) [88]. These have been shown to play a major role in hepatocyte lipid metabolism and the development of NAFLD [91]. A HFD results in reduced expression of antioxidant markers, increased lipid peroxidation, downregulated FAO genes, and increased lipogenic genes, which becomes further enhanced in Liv-SELENOM^−/−^. However, as with the inflammatory markers, overexpression of SELENOM restored all genes to their appropriate levels [88]. Together, these results suggest that SELENOM is also protective against oxidative stress and promotes lipid metabolism in hepatocytes. As we have discussed above, SELENOM is involved in the regulation of ROS, which is suggested to be important for neuroprotection in AD. Therefore, these antioxidant properties of SELENOM may be important in multiple tissues.

Further results indicated that SELENOM modulates mitochondrial stress by activating the Parkin-related mitophagy via the AMPKα1–MFN2 pathway. Mitochondrial apoptosis and mitophagy play central roles in lipotoxicity and in maintaining mitochondria quality in hepatocytes [92,93,94]. In HFD-fed mice, levels of pro-apoptotic genes were increased, and anti-apoptotic genes decreased. These mice also exhibited lower levels of mitophagy-related genes, which were further altered in HFD-fed Liv-SELENOM^−/−^ mice. These results paralleled with the in vitro model, with SELENOM overexpression returning the pro- and anti-apoptotic and mitophagy related genes to their respective levels [88]. Together these findings allude to the role of SELENOM in promoting hepatocyte survival by regulating genes involved in apoptosis and inducing mitophagy. This supports the hypothesis that SELENOM is involved in cell survival.

## 7. Outlook

Selenoproteins are critical for human function, and of the 25 identified in humans, there is still much we do not know about their role in facilitating cellular homeostasis. A better understanding of the mechanism by which Sec facilitates the function of these proteins will help guide prescription of selenium supplementation for various diseases. In this review, we have outlined what has been established for SELENOM, a small oxidoreductase widely expressed throughout the body [22,23]. We first compared structural similarities to functions. The closest structural relative to SELENOM, SELENOF, was unable to provide any functional information aside from ruling out an interaction with UGGT. However, the next closest relative is the more well-known oxidoreductase, TXN. Given that TXN is involved in disulfide bond formation, oxidoreductase assays were tested with SELENOM and found to have both TXN and GPx activity, but not TXNRD activity [25,39,40]. This infers that SELENOM oxidoreductase activity can be activated by redox stress but may also play a role in a TXN-like catalytic cycle.

Further investigation into the TXN-like behavior of SELENOM found that SELENOM might interact with TXNIP through a disulfide or selenyl-sulfide bond, modulating cellular homeostasis. This correlation has been observed in cancer in which cell growth is promoted due to high levels of SELENOM and low levels of TXNIP [49]. The opposite is true in AD, where low levels of SELENOM and high levels of TXNIP promote neuronal apoptosis due to oxidative damage [50,51]. Other interacting proteins include cellular actin, which has been specifically identified to form a disulfide (or selenyl-sulfide) bond with SELENOM [58], and Gal-1, where the binding mechanism has not been identified [54]. An imbalance of SELENOM can have deleterious effects, but what is more apparent is that SELENOM strikes a balance with its binding partners such as TXNIP; too much can cause increased cell growth and cancers, too little can cause neurodegenerative diseases. As details emerge on the interactive partners of SELENOM, we can begin to connect these interactions to observed diseases through continued fusion of mouse models, human studies, and in vitro laboratory experiments.

Combining what is known thus far, we can conclude that SELENOM is heavily involved in cellular homeostasis, through its CXXU motif. With growing technology to enable selenoprotein overexpression, outstanding questions about SELENOM and the interaction with its protein partners can be tackled. While SELENOM plays a role in certain cancers, neither reducing daily selenium intake nor having an excess of it is a straightforward remedy. Both low and high levels of selenium can be detrimental, as selenium is essential for other selenoproteins in the body that contribute to various physiological processes [1,95]. Furthermore, a direct correlation between dietary selenium levels and selenoprotein-related cancers has yet to be conclusively established. Continued efforts to unveil the functional mechanism of this protein, along with the other 24 human selenoproteins, is imperative for effective treatment of diseases.

## Data Availability

No new data were created or analyzed in this study. Data sharing is not applicable to this article.
